# Antidepressants: A content analysis of healthcare providers' tweets

**DOI:** 10.1016/j.rcsop.2023.100232

**Published:** 2023-02-10

**Authors:** Yijun Dong, Natalie M. Weir

**Affiliations:** Strathclyde Institute of Pharmacy and Biomedical Sciences, University of Strathclyde, Glasgow, Scotland, United Kingdom

**Keywords:** Antidepressants, Social media, Twitter, Content analysis, Healthcare providers

## Abstract

**Background:**

Antidepressants are the primary treatment for depression, and social support from social media may offer another support route. Whilst Twitter has become an interactive platform for healthcare providers and their patients, previous studies found low engagement of healthcare providers when discussing antidepressants on Twitter. This study aims to analyse the Twitter posts of healthcare providers related to antidepressants and to explore the healthcare providers' engagement and their areas of interest.

**Method:**

Tweets within a 10-day period were collected through multiple searches with a list of keywords within Twitter. The results were filtered against several inclusion criteria, including a manual screening to identify healthcare providers. A content analysis was conducted on eligible tweets where correlative themes and subthemes were identified.

**Key findings:**

Healthcare providers contributed 5.9% of the antidepressant-related tweets (*n* = 770/13,005). The major clinical topics referred to in the tweets were side effects, antidepressants for the treatment of COVID-19, and antidepressant studies of psychedelics. Nurses posted more tweets sharing personal experiences with commonly negative attitudes, in contrast to physicians. Links to external webpages were commonly used among healthcare providers, especially users representing healthcare organisations.

**Conclusions:**

A relatively low proportion of healthcare providers' engagement on Twitter regarding antidepressants (5.9%) was identified, with a minimal increase throughout the COVID-19 pandemic when compared to previous studies. The major clinical topics referred to in the tweets were side effects, antidepressants for the treatment of COVID-19 and antidepressant studies of psychedelics, which have been made publicly available. In general, the findings confirmed that social media platforms are a mechanism by which healthcare providers, organisations and students support patients, share information about adverse drug effects, communicate personal experiences, and share research. It is plausible that this could impact the belief and behaviours of people with lived experience of depression who may see these tweets.

## Highlights

• This is the first in-depth analysis focusing on healthcare providers' communication on Twitter relating to antidepressants, that provides insight into engagement and areas of interest.
• A low proportion of healthcare providers' engagement on Twitter about antidepressants (5.9%) was found, and pharmacists engaged less than expected.
• Twitter was often used as a platform for professional networking and education.
• Nurses communicated more about self-experiences of antidepressants and side effects, while physicians may have refrained from doing so to maintain professionalism.
• Organisational users acted more academically than individual healthcare providers by commonly sharing research citations.

## 1 Introduction

Depression is characterised by low mood, loss of interest in activities, insomnia, decreased energy, and, the major cause of mortality, suicidal thoughts.1., 2, 3 It has a global incidence of 5% with 280 million overall cases in 2019.^4^ The high prevalence and associated mortality, co-morbidities and compromised social productivity means depression is the second leading cause of disease burden.^4^^,^^5^ Pharmacological therapy is recommended in the treatment of moderate to severe depression, along with psychological treatments such as cognitive behaviour therapy.^6^^,^^7^ Most commonly prescribed antidepressants include selective serotonin reuptake inhibitors (SSRIs), serotonin-norepinephrine reuptake inhibitors (SNRIs), monoamine oxidase inhibitors and tricyclic antidepressants. However, the side effects and sometimes the unresponsiveness pose risks to patients and can be unpleasant.^8^, ^9^, ^10^, ^11^

In addition to pharmacological and psychological therapy, another critical aspect in the management of mental health is social support, in which social media has now become an influential factor alongside families and friends.^12^ Social media can provide a broad source of information, as well as online emotional support to the general population, notably to those with mental health conditions.^12^, ^13^, ^14^, ^15^, ^16^ It is reported that the primal reason for using the Internet is to acquire information for the public, with evidence suggesting that the use of internet-based social media can be used by healthcare providers as a means of sharing information in a widely accessible and cheap way, which may have an impact on patient care.^17^^,^^18^

In another aspect, social media has also presented as a platform for healthcare providers to interact with the public in an informal but more approachable way to promote public health.^18^, ^19^, ^20^, ^21^, ^22^ Social media is now performing as an influential and rapid source to disseminate information and educate medical professionals, especially in emergencies, taking the outbreak of COVID-19 as an example.^18^^,^^19^^,^^23^ Besides, with the nature of social communication platforms and their popularity, social media is also widely used for business and academic purposes such as brand marketing, career development, academic communication, and even scientific research.^18^, ^19^, ^20^, ^21^ In terms of research, social media has been used by the scientific community since its emergence.^18^, ^19^, ^20^^,^^24^, ^25^, ^26^, ^27^ Additionally, avoiding the tension of a face-to-face consultation with a physician wearing a white coat, an informal online setting may encourage the public to explore and express their feelings more genuinely and objectively.^28^^,^^29^ Overall, considering the emergence of mobile internet and that social media users account for nearly 60 % of the global population, social media has become a prominent source of public voices and communication.^26^^,^^30^

Whilst social media could be considered a place for social support during the COVID-19 pandemic and be considered a platform for open discussion, information overload and misinformation on social media poses potential mental health risks to the public.^31^^,^^32^ In general, the use of smartphones and social media is associated with increased mental distress, particularly in younger female populations, and sharing information about health can instil fear and uncertainty in populations, which was realised during the COVID-19 pandemic.^33^^,^^34^ In addition, it has also been suggested that the spread of inaccurate information may cause people to distrust medical advice, such as medicines that may be recommended for them.^31^^,^^35^ Therefore, statements by health professionals on social media could affect the public's attitude toward taking antidepressants.

Twitter, as one of the most popular microblogging platforms worldwide, allows both individual and corporate users to post content in text - up to 280 characters - to begin a dialogue, with the option of inserting other forms of digital multimedia (e.g. photos, videos).^36^ At least 500 million tweets (which is the term used for posts on Twitter) are being posted daily.^37^ The accessibility and global presence make it a notable social media platform for healthcare providers to exert influence on the general public through tweets, as well as for scientific researchers to extract opinions from a certain population.^28^^,^^29^^,^^38^ A previous study on general attitudes to antidepressants on Twitter found a low proportion of healthcare providers' referring to and discussing this.^29^ However, studies specifically exploring and reporting on healthcare providers' engagement are absent.

## 2 Aim & objective

This study aims to provide insight into the social media content on antidepressants on Twitter posted by healthcare providers, and to explore the different groups of healthcare providers' engagement and their areas of interest on this platform.

## 3 Methods & materials

This was a retrospective study on public tweets available from the microblogging and social network service, Twitter. Themes and subthemes were identified after manual inductive content analysis.

### 3.1 Data collection

A list of 115 keywords covering pharmacological classes, generic names and brand names of antidepressants was compiled based on the antidepressants approved by the Medicines and Healthcare products Regulatory Agency (MHRA) of the UK and the Food and Drug Administration (FDA) of the US (Supplement 1).^39^^,^^40^ Unofficial colloquial terms were considered unlike to be widely used by healthcare professionals and thus not included, except “antidep”, which is a commonly used abbreviation for antidepressants. Repeated searches of the listed keywords were conducted manually via Vicinitas, a Twitter analytic tool which helps to retrieve complete historical tweets.^41^ Vicinitas allows free search and output for up to 2000 tweets. Keywords exceeding this limit were purchased through Vicinitas to ensure completeness of the dataset. All the keywords were searched as text words in singular forms. The results were integrated into Microsoft Excel to remove duplicate content. The data extracted from Twitter included: nicknames, user names, bios, counts of following and followers, tweet texts, timestamps, counts of favourites and retweets, quotes, media links attached and locations.

### 3.2 Data inclusion

The eligibility criteria used to assess the tweets included: 1. Containing at least one of the keywords that refers to an antidepressant(s) or the term ‘antidepressant’; 2. Written in English; 3. Posted between a 10-day period between 14th June 2022 and 23rd June 2022; 4. Containing original text; 5. Posted by a user who self-identified as a healthcare provider. Several preliminary searches determined a 10-day span would be feasible considering the scale of data and the capability of manual analysis. Posts on Twitter are generally categorised into tweets (including those that quoted another tweet), retweets and replies. For this study, only tweets and replies were included, since retweets are identical reposts and thus were considered duplicate content.

The inclusion/exclusion criteria were applied within Microsoft Excel, by authors manually reviewing the textual data. Microsoft Excel allowed screening and excluding data easily according to our criteria 1–4. For criterion 5, user profiles (names and bios) associated with the identified tweets were assessed for eligibility, whereby tweets posted by healthcare providers were eligible and categorised by their roles byYD. The definition of “healthcare providers” (Table 1) was adapted from the version defined by Lee et al. which was previously used to conduct a Twitter analysis of healthcare providers.^42^ It was expanded in this study to”relevant healthcare professionals, providers and students”, who were considered people or organisations which the general public may expect to be more knowledgeable about healthcare, such as physicians (including psychiatrists), nurses, pharmacists, psychologists, researchers, medical students, and organisations in medical fields and other allied professionals (e.g, therapists, dietitians). Where there was ambiguity over someone's eligibility due to dubious expression over their role, a second researcher (NW) was consulted. If it was not certain that they were a healthcare provider, i.e., they did not use definitive terminology such as ‘pharmacist’ or ‘medic’, they were not included in the analysis. The content of the tweet text had not been considered in this stage to avoid bias.

**Table 1:** Descriptions and Examples of Categories of Healthcare Providers Identified: This table explained the classification of healthcare providers in this study and listed some examples of their presentations. Identifying information was adapted.

Category	Description	Example Bio (partially adapted)
Physicians	Physicians, psychiatrists, doctors from various clinical subjects, dentists, ophthalmologists	“MD […] #physiatrist […]”, “Maternal-Fetal Medicine Physician”, “MBBS (GMC, […])”, “Doctor | Special interest in Long Covid […]”, “Clinician-scientist […]”
Nurses	Nurses, nurse practitioners, registered nurses, retired nurses	“Mom, nurse, wife, daughter, and sister. […]”, “STICU Nurse. […]”, “28. Labor & delivery RN. […]”
Pharmacists	Pharmacists, mental health pharmacists, clinical pharmacists from various clinical subjects	“PharmD/writer w/30y exp.Infect Diseases. […]”, “Retail Pharmacist”, “Mental Health Clinical Pharmacist Practitioner […]”
Psychologists	Psychologists, clinical psychologists	“Dual national,CPsychol, accidental academic. […]”, “A retired developmental psychologist […]”
Medical Students	Medical students, MD and PharmD candidates, students of biomedical areas	“PGY-1 in rural/full-spectrum family med […]”, “[…] | Med student | […]”, “Internal medicine residency applicant”
Organisations	Hospitals, clinics, healthcare businesses, academic groups, journals, medical information providers, charities	“Real vitamins for physical and mental health. […]”, “Original research in physiology with an emphasis on adaptive and integrative mechanisms | An @APSPhysiology journal”, “ACTIV-6 is a research study testing repurposed medications to understand if they can help people with mild-to-moderate COVID-19 feel better faster.”
Researchers	Individual researchers specified in biomedicine, mental health, public health areas	“COVID scientist; Associate Professor of Psychiatry @[…]”, “Training psychiatrist/research fellow @[…]”, “Researcher in Pharmacognosy, Pharmacology & Pharmacy. […]”
Others Allied Professionals	Therapists, dieticians, nutritionists, midwives, social workers, hygienists, health care educators, unspecified professionals	“Social worker in […]”, “[…] Mental Health Therapist […]”, “Health Care Provider, […]”, “NHS midwife, […]”, “Registered Dietitan, Cannabis practitioner, […]”

### 3.3 Content analysis

The data were then imported into NVivo, a qualitative data analysis software often used for content analysis. Each tweet in the dataset was analysed and labelled with correlative themes and subthemes. Ten per cent of tweets were sampled randomly and tentatively coded to define appropriate themes and subthemes by YD. To control the quality of the coding, another researcher NW coded these 10% of initial tweets independently. The themes and subthemes identified were developed into a coding framework which was agreed on by both researchers. The remaining tweets were coded by YD, and a cross-comparison of different users was conducted after the analysis.

### 3.4 Ethical considerations

All the data collected from Twitter was publicly available for viewing. Conducting academic research on the general public's tweets, according to the privacy policy of Twitter, has been consented to by all Twitter users.^43^ All the identifying information (e.g. user profile, coordinates) has been deleted or adapted to maintain privacy and anonymity, and none of the information was or will be used for anything other than academic research purposes. Ethical approval was exempted by the Strathclyde Institute of Pharmacy and Biomedical Sciences Ethics Committee as this study only involved publicly available historical data.

## 4 Results

### 4.1 Data collection

**Fig. 1:**
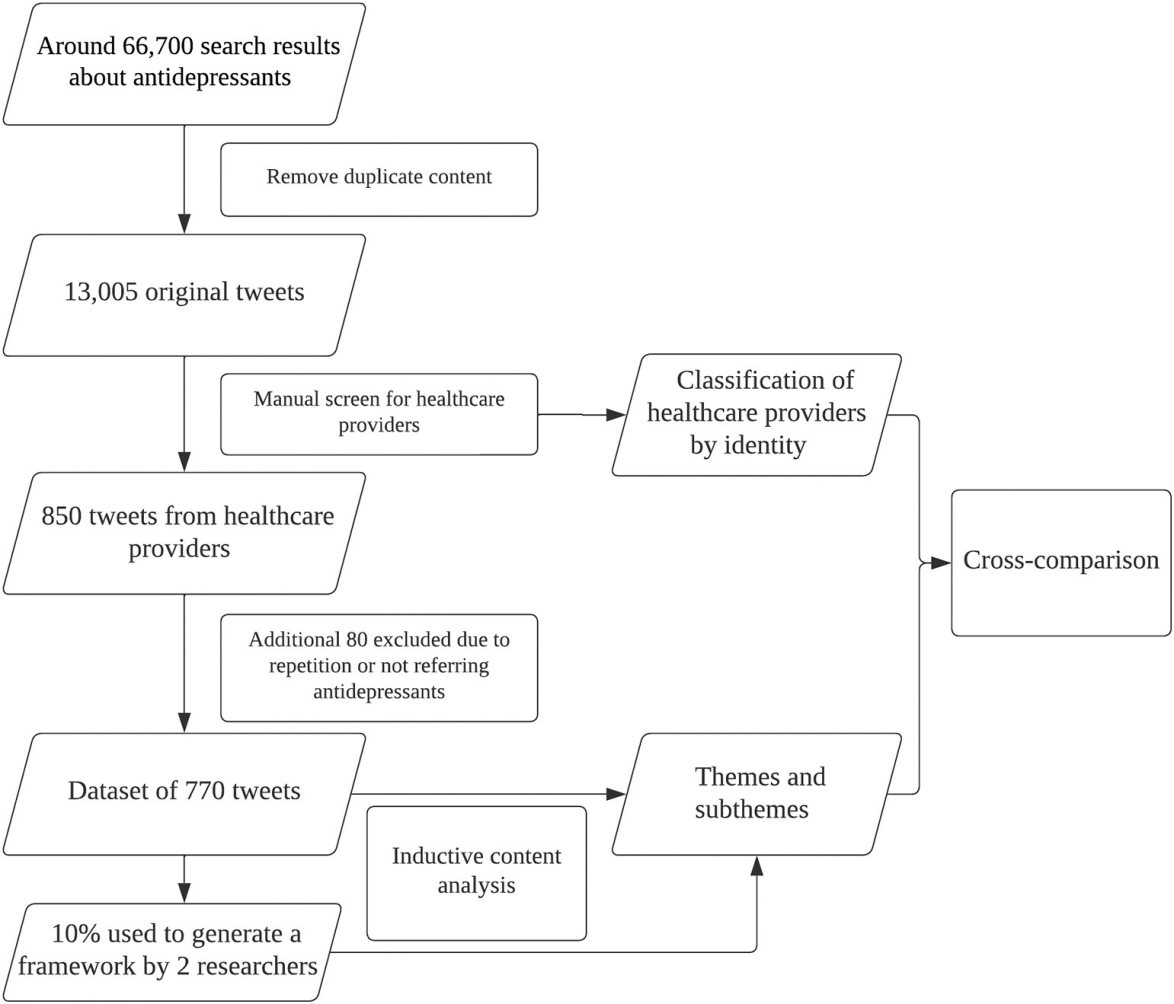
Flowchart of Data Process: Tweets within a 10-day period were collected through multiple searches with a list of keywords within Twitter and filtered with several certain inclusion criteria, including a manual screening to identify healthcare providers. A content analysis was conducted on those eligible tweets where correlative themes and subthemes were identified.

A total of approximately 66,700 tweets were identified from the search strategy, and 13,005 original tweets remained after filtering following the aforementioned inclusion criteria (e.g. removal of retweets and non-English tweets). Only an approximate count of 66,700 was available as Vicinitas only offers approximate total searches before removing retweets. Thereafter, it was identified that 850 tweets were considered posted by healthcare providers. Meanwhile, 80 of them were considered ineligible (repetition or the keywords not referring to antidepressants) and thus were excluded from the analysis. The final dataset consisted of 770 (5.9%, 770/13,005) tweets posted by 562 identified healthcare providers (Fig. 1), 49.4% (380/770) of which were tweets and 50.6% (390/770) were replies to tweets.

The most frequent keyword mentioned by healthcare providers was “antidepressants” (*n* = 200, 26.0%) followed by “SSRI” (*n* = 82, 10.6%), “Effexor” (*n* = 56, 7.3%), “Zoloft” (*n* = 34, 4.4%), “sertraline” (n = 34, 4.4%), “bupropion” (n = 34, 4.4%), “fluoxetine” (*n* = 29, 3.8%), and “Paxil” (n = 29, 3.8%).

### 4.2 Roles of identified healthcare providers

**Fig. 2:**
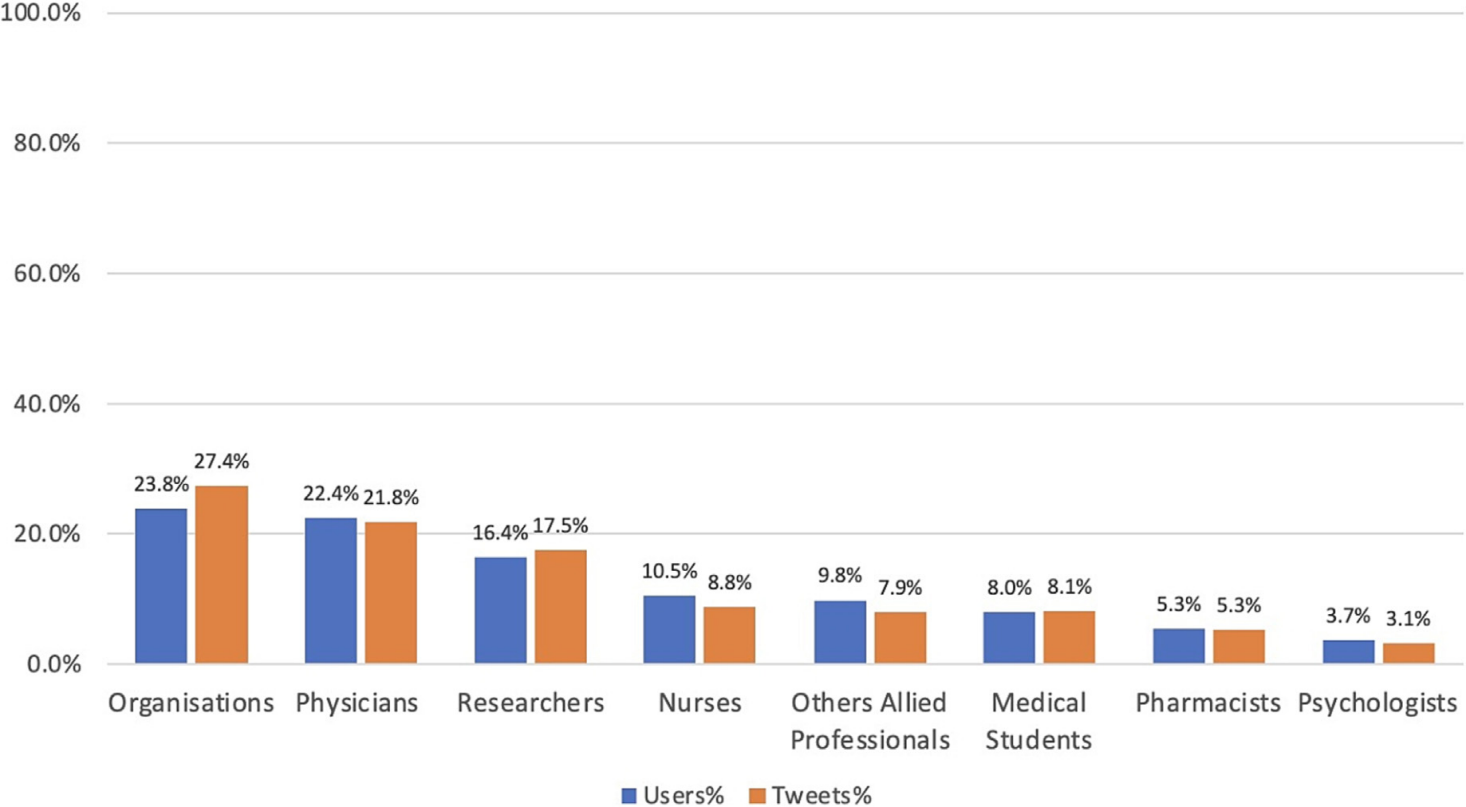
Contributions of Users and Tweets by Categories: Organisational users were the most active group, whereas pharmacists engaged less.

Among the 770 identified tweets from healthcare providers, the healthcare providers were categorised into several groups (Fig. 2). These tweets were most commonly posted by representatives of organisations (*n* = 211, 27.4%), followed by physicians (*n* = 168, 21.8%), researchers (*n* = 135, 17.5%), nurses (*n* = 68, 8.8%), medical students (*n* = 62, 8.1%), other allied professionals (*n* = 61, 7.9%), pharmacists (*n* = 41, 5.3%) and psychologists (*n* = 24, 3.1%).

## 5 Content analysis

### 5.1 Overview

The dataset of 770 tweets was generally classified into clinical aspects (*n* = 594, 77.1%) and non-clinical aspects (*n* = 138, 17.9%), while 38 tweets (5.0%) were considered unclassifiable due to the lack of context. Table 2 presents the key themes identified with exemplar tweets. Among 229 tweets (29.7%) that included links, 90 (11.7%) cited academic studies and 79 (10.2%) cited website articles. The rest linked to medical information websites (including clinical service websites, *n* = 23, 3.0%), other tweets (*n* = 21, 2.7%), business websites (*n* = 12, 1.6%) and videos (*n* = 4, 0.5%). The most notable subthemes were listed as follows.

**Table 2:** Examples of Major Themes and Subthemes: Some typical tweets of major themes and subthemes and their prevalence are presented where identifying information was adapted.

Area	Theme (Number of tweets, %)	Typical tweets (partially adapted)
Clinical aspects n = 594, 77.1%	Side effects *n* = 134, 17.4%	“@[usernames] I use cymbalta a lot for CIPN for my Pts. I’'ve learned the hard way that it has to be tapered. Can also cause WICKED headache on withdrawal” “@[usernames] Lexapro was good for me but the only bad thing about it that I experienced was SEVERE decrease in libido”
	Antidepressants for other uses *n* = 128, 16.6%	“@[usernames] The original idea to use fluvoxamine to treat COVID was because of the sigma1 agonist action. https://t.co/daqDoZj2xx” “@[usernames] Same! Well, I take Nortriptyline for chronic migraines but that's a different story [crying with laughter emoji]”
	Other antidepressive substances *n* = 91, 11.8%	“What is a patient's experience really like with ketamine treatment? We spoke with a member of the Osmind Patient Community about their experience with the novel antidepressant treatment and the resulting effect on his mental health. https://t.co/Pk0NdyyE0O” “5. 5-Hydroxytryptophan (5-HTP) 5-HTP is a chemical derived from the seeds of an African plant known as Griffonia simplicifolia, which has been studied for its antidepressant effects on people with depression or anxiety disorders.”
	Efficacy of antidepressants *n* = 47, 6.1%	“More evidence of placebo effects in antidepressant treatments. @[usernames] https://t.co/Vq8tBN6xeR” “7/10 Taking an SSRI helps me to feel well and to feel like me, the best version of me. It's not a cure, I'm still a slightly anxious person and I need to using strategies to manage it, but it makes a big difference to my ability to cope and it means that I don't feel stressed”
Non-clinical aspects n = 138, 18.0%	Metaphorical and rhetoric use *n* = 33, 4.3%	“My wife (…) has become my antidepressant for life.” “BEY-FLOW-XETINE my favorite antidepressant [love heart eyes emoji] https://t.co/QutdgU7ofH”
	Names of antidepressants n = 23, 3.0%	“@[usernames] Elavil sounds like a medieval enchantress” “@[usernames] Celexa for a girl, Prozac for boy.”
	Commercial advertisements *n* = 19, 2.5%	“Bupropion HCL 150 mg-Extended release coated tablets: #Bupropion, an #antidepressant with moderate noradrenergic & dopaminergic action for the treatment of depression and symptoms produced by smoking abstinence. [red circle emoji]: #Depression #smokingabstinence —[letter emoji] info@tajpharma.com #TajPharma https://t.co/ausYCiMaog” “CHEAPEST Generic #Duloxetine WITHOUT INSURANCE ONLY $0.43 A PILL #Duzela is used for the symptomatic relief of #majordepressivedisorder & #generalizedanxietydisorder. Also used to treat #neuropathicpain https://t.co/2lYwdKZUxO”
Unclassifiable - unclear due to the lack of context *n* = 38, 4.9%		“@[usernames] Pristiq” “@[usernames] Which SNRI u used ???”
Additional aspects	Self-experience *n* = 97, 12.6%	“@[usernames] Yeah I just started on fluoxetine a few weeks ago… It's definitely not fun!” “@[usernames] That so resonates with me, I've been on a small dose of Sertraline for 2 years and with that, exercise and CBT, I feel like myself again…#anxiety”
	Supporting patients *n* = 74, 9.6%	“@[usernames] Not so. If intolerable withdrawal symptoms lasting more than a couple of weeks from cold turkey, consider reinstatement of a teeny dose, such as 1 mg if you were taking 20 mg escitalopram. This can stop withdrawal. You'd taper off later. FYI Protracted withdrawal can last years.” “@[usernames] Might be helpful to bring notes about your negative side effects and/or disappointing treatment outcomes with fluoxetine. They should listen. They may suggest other options based on your symptoms. This is an entirely reasonable conversation to have. Good luck!!! [two green love heart emojis]”
	Perceived prescribers lack of knowledge *n* = 15, 1.9%	“@[usernames] Sweating is also a sign that your #antidepressant dosage is too high. Your prescriber likely doesn't have a clue about this.” “@[usernames] I discovered this the hard way. A psychiatrist ‘tapered’ me off 40mg of Citalopram over 10days. My severe and protracted withdrawal symptoms were misdiagnosed as relapse. More drugs prescribed. Eventually I found help online. Doctors were clueless 1/2”

### 5.2 Clinical aspects of antidepressant

A total of 594 tweets (594/770, 77.1%) referred to clinical aspects of antidepressants. Some of the tweets mentioned more than one clinical aspect, so were coded more than once. Thus the 594 tweets were coded a total of 644 times. More than half of the codes associated with the following three major themes.

The most prevalent theme about antidepressants was side effects (134/644, 20.8%) with a prominent subtheme of withdrawal symptoms (38/134, 28.4%). Other subthemes were sexual dysfunction and decreased libido (9/134, 6.8%); heatstroke, increased heat sensitivity and hyperthermia (7/134, 5.2%); insomnia (7/134, 5.2%); violence and homicidal ideation (7/134, 5.2%); weight gain (6/134, 4.5%); nausea and vomiting (5/134, 3.7%); suicidal ideation (5/134, 3.7%); and 29 other minor side effects (37/134, 27.6% in total), with 13 cases (13/134, 9.7%) of unspecified unpleasant experiences.

Antidepressants for other uses (128/644, 19.9%) was the second most common theme, in which the subthemes were antidepressants (e.g. fluvoxamine) used in COVID-19 treatment (34/128, 26.6%), smoking cessation (13/128, 10.1%), pain (12/128, 9.4%), anxiety (8/128, 6.2%), inflammation (7/128, 5.5%) and panic disorders (6/128, 4.7%). The rest were referring to 30 other uses (48/128, 37.5% in total).

The third major theme was other antidepressive substances (91/644, 14.1%). The majority referred to the antidepressant effect of psychedelics (64/91, 70.3%), especially ketamine (20/91, 22.0%), esketamine (15/91, 16.5%) and psilocybin (13/91, 14.3%). There were 16 tweets (16/91, 17.6%) referring to another 13 chemicals under development and research, 7 (7/91, 7.7%) relating to probiotics, 3 (3/91, 3.3%) to foods, and 1 (1/91, 1.1%) to soil microbes.

Other notable clinical aspects included the effectiveness of antidepressants (47/644, 7.3%), interactions (29/644, 4.5%), other medical issues (e.g. discussing academic studies involving antidepressants, 29/644, 4.5%), sharing personal stories (28/644, 4.3%), new studies on antidepressants (23/644, 3.6%), antidepressant use during pregnancy (21/644, 3.3%), and general public health issues (e.g the stigma of depression, 21/644, 3.3%).

### 5.3 Non-clinical aspects

The total number of classified non-clinical codes was 138 (17.9%). The most common subtheme was referring to antidepressants in a rhetorical form (33/138, 23.9%), including indicating music (4/138, 2.9%) or sunshine (3/138, 2.2%) being an antidepressant for them. Another popular theme was regarding the names of antidepressants (22/138, 15.9%). Most contents were respective answers to a tweet asking “You have to name your child after a prescription drug, what are you going with”. There were 19 tweets (19/138, 13.8%) marked commercial advertisements, 17 (17/138, 12.3%) talking about antidepressants in daily lives, 8 (8/138, 5.8%) regarding medical insurance issues, 7 (7/138, 5.1%) quizzes and the others were miscellaneous issues such as academic conference publicity (6/138, 4.3%), price issues (6/138, 4.3%) and antidepressants in veterinary use (5/138, 3.6%).

### 5.4 Additional aspects

In total, 97 tweets (12.6%) were posted by healthcare providers sharing their self-experience as a patient. Most of them (48/97, 49.5%) held negative attitudes, while 31 (31/97, 32.0%) were positive. The others were either neutral (9/97, 9.3%) or it was not possible to decipher their attitude (9/97, 9.3%). There were 74 tweets (9.6%) that considered supporting patients by giving professional advice (30/74, 40.5%), explaining mechanisms (23/74, 31.1%) or emotionally supporting (21/74, 28.4%). There were also 15 tweets (1.9%) perceiving prescribers lacked knowledge regarding antidepressants.

### 5.5 Cross-comparison of users and tweets

A cross-comparison was conducted (Table 3). When comparing different healthcare providers' tweets, it appeared that nurses tweeted more about side effects than other groups. Additionally, nurses posted a disproportionately high fraction of content on their personal experiences, while physicians tended not to. Organisational users used more links in tweets than individuals and contributed to the majority of links to external webpages and commercial links.

**Table 3:** Cross-comparison of Users and Tweets of Major Themes: A cross-comparison by themes and different groups of healthcare providers was conducted. Some prominent points were found in this table. First, nurses talked more about side effects than other groups. Second, nurses posted a disproportionately high fraction of content on their personal experiences, while doctors showed the opposite. Third, organisational users used more links in tweets than individuals and contributed to the majority of academic citations and commercial links.

Area	Theme	Physicians	Medical Students	Nurses	Organisations	Others Allied Professionals	Pharmacists	Psychologists	Researchers	Total
Count of tweets posted		168(21.8%)	62(8.1%)	68(8.8%)	211(27.4%)	61(7.9%)	41(5.3%)	24(3.1%)	135(17.5%)	770(100%)
Clinical aspects	Overall	114(19.2%)	43(7.2%)	55(9.3%)	177(29.8%)	46(7.7%)	26(4.4%)	22(3.7%)	111(18.7%)	594(100%)
	Side effects	22(17.7%)	13(10.5%)	24(19.4%)	26(21.0%)	14(11.3%)	8(6.5%)	4(3.2%)	13(10.5%)	124(100%)
	Antidepressants for other use	33(26.8%)	7(5.7%)	8(6.5%)	29(23.6%)	6(4.9%)	7(5.7%)	5(4.1%)	28(22.8%)	123(100%)
	Other antidepressive substances	17(19.3%)	2(2.3%)	0(0.0%)	50(56.8%)	3(3.4%)	0(0.0%)	1(1.1%)	15(17.0%)	88(100%)
Clinically irrelevant aspects		42(30.4%)	13(9.4%)	9(6.5%)	33(23.9%)	12(8.7%)	10(7.2%)	2(1.4%)	17(12.3%)	138(100%)
Unclassifiable		13(34.2%)	6(15.8%)	4(10.5%)	1(2.6%)	3(7.9%)	5(13.2%)	0(0.0%)	6(15.8%)	38(100%)
Links	Overall	24(10.6%)	7(3.1%)	6(2.6%)	135(59.5%)	7(3.1%)	5(2.2%)	5(2.2%)	38(16.7%)	227(100%)
	Academic studies	13(14.4%)	1(1.1%)	1(1.1%)	46(51.1%)	1(1.1%)	2(2.2.%)	1(1.1%)	25(27.8%)	90(100%)
	Web article	7(8.9%)	0(0.0%)	0(0.0%)	57(72.2%)	4(5.1%)	0(0.0%)	3(3.8%)	8(10.1%)	79(100%)
	Business websites	0(0.0%)	1(8.3%)	0(0.0%)	11(91.7%)	0(0.0%)	0(0.0%)	0(0.0%)	0(0.0%)	12(100%)
Self-experience	Overall	10(10.3%)	14(14.4%)	30(30.9%)	0(0.0%)	19(19.6%)	7(7.2%)	4(4.1%)	13(13.4%)	97(100%)
	Negative	2(4.2%)	9(18.8%)	18(37.5%)	0(0.0%)	9(18.8%)	4(8.3%)	1(2.1%)	5(10.4%)	48(100%)
	Positive	7(22.6%)	2(6.5%)	6(19.4%)	0(0.0%)	6(19.4%)	2(6.5%)	3(9.7%)	5(16.1%)	31(100%)
	Neutral	0(0.0%)	2(22.2%)	4(44.4%)	0(0.0%)	0(0.0%)	0(0.0%)	0(0.0%)	3(33.3%)	9(100%)
Supporting patients		16(21.9%)	10(13.7%)	10(13.7%)	9(12.3%)	9(12.3%)	5(6.8%)	2(2.7%)	12(16.4%)	73(100%)

## 6 Discussion

### 6.1 Overview

This study has several principal findings. First, the engagement of the healthcare providers was noticeably low, especially for pharmacists. Second, the major clinical topics related to side effects, antidepressants for other uses (e.g. fluvoxamine for the treatment of COVID-19) and other antidepressants (e.g. studies of psychedelics). Third, the prevalence of sharing personal experiences presented a contrast between physicians and nurses. Fourth, links to external webpages were commonly used among healthcare providers, notably by organisational users.

### 6.2 Healthcare providers' engagement on Twitter

The engagement of the healthcare providers was significantly lower than those of the general public (5.9% versus 94.1%), and pharmacists surprisingly contributed comparably less than most other healthcare providers (5.3%). A prior study by de Anta et al. found healthcare providers contributed only 3.5% of the general tweets related to antidepressants, according to the data collected between January 2019 and October 2020.^29^ Compared to that, the result of 5.9% from this study suggests a minimal increase. However, given the methodological difference in eligibility and definition, such a rise may be considered insignificant. Since the COVID-19 pandemic in early 2020, the global burden of mental health has been exacerbated by the spread of the virus, social isolation, industrial lockdowns and diminished mental health services. It is estimated a 27.6% increment in the prevalence of the general population with major depressive disorder, along with significant rises in anxiety, post-traumatic stress disorder (PTSD) and other mental health conditions.^32^^,^^44^, ^45^, ^46^ It is suggested that Twitter can be used as a platform for patient education not only to promote mental health through online social support but can also help to ease the stigma of psychiatric disorders.^21^^,^^47^^,^^48^ During the pandemic, it was found that the public was more dependent on social media to receive and share information with peers.^44^ A previous literature review suggested that although the optimal balance of digital and conventional healthcare was still to be explored, social media has already become a ubiquitous and versatile tool in healthcare.^18^ Despite this, in this study, less than 10% of the identified tweets (74/770) involved healthcare providers supporting patients. Though it could have been anticipated that healthcare providers may take more part in social media to support mental ill health considering its well-established negative impact on mental health and the impact on healthcare accessibility, these results indicate their engagement with discussion on antidepressants only minimally increased throughout the pandemic.

Furthermore, this study found that pharmacists, who are considered experts on medicines including antidepressants, contributed only 5.3% of tweets among healthcare providers. The discrepancy between the population of pharmacists with other groups of professionals is a possible contributing factor. The number of pharmacists was approximately one-third of the number of physicians in the US, and below one-fourth of the number of physicians in the UK.49., 50, 51., 52. However, while pharmacists have a role to play in the management of somatic diseases, their role in supporting patients in mental health is less clear. Elewa et al. and Gillani et al. suggested pharmacists' involvement may improve the outcomes of pharmacotherapy of anticoagulants, but a systematic review by Brown et al. made a contrary conclusion in depression management.^53^, ^54^, ^55^ It is suggested that pharmacists may exert more influence in mental health care to achieve a better quality of pharmacotherapy. Effective approaches include detecting early-stage mental health illnesses, collaborative work with multidisciplinary teams and taking active interventions to improve adherence.^56^ However, several studies have revealed the gap between the current and potential role of pharmacists in the care of mental health, despite their positive willingness.^57^, ^58^, ^59^ Meanwhile, Benetoli et al. also found a void in implementing social media in pharmacy services for community pharmacists.^60^ To conclude, the lack of pharmacists' engagement on Twitter may be due to their lesser role in mental health care and their limited engagement with social media platforms.

### 6.3 Prevalent clinical themes

The most popular clinical topic of antidepressants on Twitter was their side effects, predominantly withdrawal symptoms. Besides a wide range of side effects, more than half of the patients may experience symptoms like dizziness, irritability, restlessness, sweating and tiredness after abrupt discontinuation of antidepressants or missing doses.^9^^,^^61^^,^^62^ Such withdrawal symptoms were underestimated in the past decades since they are likely mistaken for other side effects or relapses of depression.^61^^,^^62^ Nevertheless, NICE recently released a new version of guidelines for adult depression in June 2022, which updated and offered significantly more information on withdrawal symptoms, suggesting greater awareness in general.^63^ Another proportion of tweets focused on the efficacy of antidepressants. There were views indicating that the efficacy of antidepressants is overestimated due to the placebo effect and the subjective measurement of the symptoms and effects in mental health.^64^, ^65^, ^66^, ^67^ Since social media also acts as a source of information, such messages presented by healthcare providers on Twitter might communicate a more negative image of antidepressants to patients. It is possible that access to such information may hinder newly diagnosed patients from initiating pharmacotherapies.

Often, tweets referred to antidepressants for other uses and novel antidepressants. The most popular topic among these was antidepressants used for COVID-19. Fluvoxamine was found promising in the early treatment of COVID-19 in reducing hospitalisation and alleviating the impact of long-COVID symptoms through its anti-inflammatory effect, with several papers cited in the tweets.^68^, ^69^, ^70^, ^71^ The antidepressant effects of psychedelics, such as ketamine, esketamine (S- form of ketamine) and psilocybin, were also referred to, with evidence emerging that they can provide effective rapid-acting therapy for treatment-resistant depression through novel pathways.^72^, ^73^, ^74^, ^75^ It is noteworthy that esketamine nasal spray (Spravato) was approved by the FDA, Washington in March 2019, and Scottish Medicines Consortium, Scotland in August 2020 for treatment-resistant depression, but refused by NICE, England for the third time in May 2022 due to clinical and cost concerns, which also appeared in the Twitter dialogues.^76^, 77., 78. It is apparent that Twitter is being used as a platform to disseminate information associated with novel uses of antidepressants, with evidence shared within research communities and to the general public.^18^^,^^21^

### 6.4 Supporting patients

There were also some tweets identified offering support to patients, in which pharmacists contributed a relatively low proportion (*n* = 5/74). Common forms of support consisted of giving professional advice on easing adverse effects, providing recommendations in choosing medication, explaining mechanisms of action and offering emotional support (e.g., empathising and encouraging). Prior studies have revealed a negative correlation between social support and depression.^12^ Such online patient support could be a possible help in improving symptoms while enhancing the efficacy of pharmacotherapy. It could be a potential opportunity for community pharmacists to exert more influence in psychiatry by disseminating evidence-based information and offering emotional support on social media.^79^

### 6.5 Sharing self-experiences as a patient

Many tweets mentioned self-experience with antidepressants as a patient. Particularly, it was more common for nurses to share self-experiences, which were predominately negative, whereas fewer physicians shared predominantly positive insights. A doubled prevalence of depressive symptoms in nurses than in the general population might explain why they referred to personal experience on antidepressants, as well as their side effects, more often.^80^^,^^81^ It was also reported that social media played an important role in releasing stress for healthcare providers, especially during the isolation period of the COVID-19 pandemic.^18^ Besides, Kocemba et al. suggested that physicians' professionalism may be compromised if they publicly reveal personal details.^82^ It is also recommended by the American Medical Association to separate professional and personal information on social media, which might explain cultural reasons why physicians shared their personal experiences of antidepressants less.^83^

### 6.6 Citation of articles

It is found that links to external webpages such as peer-reviewed journals and website articles were widely cited in healthcare providers' tweets, and more often by organisational users than individuals. Many of the citations were used for academic purposes where users were publicising new studies to their followers. This could be due to social media being widely used as a portal for professional education.^18^^,^^23^ Meanwhile, by sharing new studies or their own works, social media offers a route for networking with their peers in similar areas.^18^ Additionally, Ozkent found that more exposure to social media may lead to a higher citation rate of an article.^84^ Unsurprisingly, organisational accounts contributed the majority of citations, as they were created to represent the collective group in a professional stance. For individuals, it may be hard to define the boundary between professional and personal uses of social media.^85^ As an example, disclaimers like “tweets are not medical advice” were often found in individual healthcare providers' bios. As previous research suggested that almost half of the medical content in tweets can be considered inaccurate, healthcare providers on Twitter may use citations to help to indicate the reliability of their tweets and claims.^29^^,^^86^

### 6.7 Strengths

To the authors' knowledge, this study is the first content analysis focusing on healthcare providers' engagement on Twitter relating to antidepressants and provides an in-depth analysis, as previous studies have offered less detail.^29^ A comprehensive search was conducted to minimise sampling bias in data collection, in which the keywords covered both generic and brand names of the complete list of licensed antidepressants by the authorities of two major English-speaking countries in Twitter, the US and the UK. In addition, a validated framework was used to categorise healthcare providers.^42^

### 6.8 Limitations

Certain limitations remain in this study. First, due to the scale of this study, a limited span of 10 days was adopted, thus the numbers became low when cross-comparing subgroups of healthcare providers. It is also possible that cultural and significant events during the 10-day time span could have influenced the results. To the authors' knowledge, there were no events specific to mental health and depression which could have obviously impacted the search (e.g., mental health awareness campaigns). Second, the inclusion of healthcare providers was based on self-stated information, which may not be accurate, and it also requires the subjective interpretation of the researchers to determine eligibility. In addition, it is plausible that a significant portion of healthcare providers, professionals and students do not disclose this role on their Twitter profile. The extent to which this is the case is unknown, so it is likely our results are an underrepresentation. Third, the demography of data was limited by the users of Twitter and no other social media platforms were explored, and a proportion of tweets are protected by tweet privacy policies when individuals make them private to the public, hence inaccessible.^87^ Besides, the influence of Twitter may be altered by the impact of other social media platforms, such as Facebook, YouTube, Instagram, and especially by the increase in Tik Tok usage.^17^ However, the nature of Twitter's worldwide popularity and the content primarily in text still renders it a useful platform to be studied.^88^ Further studies may consider implementing technologies like machine learning to include a broader range of data or to develop novel algorithms to analyse other graphical and video platforms.

## 7 Conclusion

A relatively low proportion of healthcare providers' engagement on Twitter about antidepressants (5.9%) was found, with a minimal increase when compared to previous studies. In general, the findings confirm that social media platforms are a mechanism by which healthcare providers, organisations and students share information about adverse drug effects, communicate personal experiences, share research, and support patients,. However, it is unclear why there was limited pharmacists' engagement, and future research may wish to explore interprofessional differences in social media usage. The major clinical topics referred to in the tweets were side effects, antidepressants for the treatment of COVID-19, and antidepressant studies of psychedelics. It is plausible that this could impact the belief and behaviours of people with lived experience of depression who may see these tweets; however, the extent of which is unclear and future research is needed to explore the impact on the public.

## Declaration of Competing Interest

Both authors declare that they have no conflicts of interest. This research was not funded.

## Appendix A Supplementary data

Supplement 1: List of 115 Keywords Used in Data Collection Image 1
