# Supplementary material for: Antidepressants: A content analysis of healthcare providers' tweets
Source: Explor Res Clin Soc Pharm. 2023 Feb 10;9:100232. doi: 10.1016/j.rcsop.2023.100232 (PMC9976573; doi:10.1016/j.rcsop.2023.100232)
Supplement: Supplementary file 1 — Supplement 1: List of 115 Keywords Used in Data Collection [file mmc1.docx]

**Supplement 1 List of 115 Keywords Used in Data Collection**

antidepressant, antidep, selective serotonin reuptake inhibitors, serotonin norepinephrine reuptake inhibitor, monoamine oxidase inhibitors, SSRI, SNRI, agomelatine, amitriptyline, citalopram, clomipramine, dosulepin, doxepin, duloxetine, escitalopram, flupentixol, fluoxetine, fluvoxamine, imipramine, isocarboxazid, lofepramine, mianserin, mirtazapine, moclobemide, nortriptyline, paroxetine, phenelzine, reboxetine, sertraline, tranylcypromine, trazodone, trimipramine, venlafaxine, vortioxetine, vilazodone, levomilnacipran, desvenlafaxine, amoxapine, maprotiline, desipramine, protriptyline, nefazodone, bupropion, selegiline, esketamine, brexanolone, Valdoxan, Cipramil, Celexa, Cymbalta, Prothiaden, Sinepin, Sinequan, Cipralex, Lexapro, Fluanxol, Olena, Oxactin, Prozac, Prozep, Faverin, Lomont, Zispin, Remeron, Manerix, Allegron, Pamelor, Seroxat, Paxil, Pexeva, Nardil, Edronax, Lustral, Zoloft, Parnate, Molipaxin, Desyrel, Surmontil, Alventa, Amphero, Depefex, Efexor, Effexor, Foraven, Majoven, Politid, Sunveniz, Tonpular, Venadex, Venaxx, Vencarm, Venlablue, Venladex, Venlasoz, Venlalic, Vensir, ViePax, Brintellix, Trintellix, Viibryd, Fetzima, Pristiq, Khedezla, Asendin, Elavil, Ludiomil, Norpramin, Tofranil, Vivactil, Serzone, Wellbutrin, Emsam, Marplan, Spravato, Zulresso.
